# Unfolding the roles of resveratrol in p53 regulation

**DOI:** 10.18632/oncotarget.26185

**Published:** 2018-10-02

**Authors:** Rafaela Muniz de Queiroz, Carol Prives

**Affiliations:** Carol Prives: Department of Biological Sciences, Columbia University, New York, NY, USA

**Keywords:** p53, aggregation, resveratrol, protein refolding, cancer

Prion-like disorders, which have as their main characteristic the formation of aggregates consisting of amyloid fibrils and oligomers, have been well studied in the context of neurodegenerative diseases, most famously Alzheimer’s. Yet prion-like characteristics may play a role in cancer as well. For example, the *TP53* gene encodes a protein with multiple roles in tumor suppression that is frequently mutated in many forms of human cancer and some missense mutant p53 proteins can form prion-like aggregates. In the 90’s researchers demonstrated that both wild-type and mutated p53 proteins form high molecular weight complexes that are highly insoluble in physiological conditions [6]. Later it was shown that p53 aggregates found in cancer cells have properties that are similar to alpha-synuclein and beta-amyloid aggregates, both related to neurodegenerative diseases [4]. Indeed, there is increasing evidence that formation of aggregates is one of the mechanisms leading to p53 loss-of-function in cancer cells [8] and such aggregates are even associated with chemoresistance [11].

Resveratrol, a polyphenol derived from many plant species, has emerged as a promising antitumor agent. This natural compound exhibits cytotoxic effects against tumor cells and one of the mechanisms by which resveratrol induces cell death is through activation of p53 [3].

Recently in this journal the Silva group have investigated possible interactions between resveratrol and the DNA-binding domain of p53 as a mechanism for modulation of p53 aggregation [2]. They have provided evidence indicating that resveratrol interacts with the core DNA-binding domains of both wild-type p53 and a common tumor-derived mutant (R248Q) *in vitro* and that such interaction reduces aggregation of the p53 core domain. The authors demonstrate that treatment with resveratrol decreases the amount of p53 oligomers in human cancer cells harboring the gain-of-function mutant p53 proteins (R280K and R248Q). They also provide data that mutant p53 in xenografted MDA-231 cell-derived tumors from mice that were treated with resveratrol is less reactive with an antibody that specifically recognizes mutant p53 aggregates. Relatedly, they show that both cell proliferation and motility of breast cancer cells are reduced upon resveratrol treatment. Although additional evidence is warranted before reaching a firm conclusion that resveratrol can be used to mitigate the oncogenic functions of mutant p53, the data provided by Ferraz da Costa and colleagues is very intriguing indeed. Prior to this study, evidence linking resveratrol and p53 activation indicated an indirect effect of the compound by inducing post-translational modifications of the p53 protein leading to its stabilization and activation. That there is a direct interaction between resveratrol and p53 as reported by Ferraz da Costa et al. (2018) adds another layer of complexity to its role in p53 modulation.

Many questions can be posed as a result of this study. First, since the authors’ evidence points to a direct interaction of the drug with the p53 specific DNA binding domain, it is reasonable to investigate how the transcriptional activity of wild-type and mutant p53 are affected by their binding to resveratrol. For example, can this agent impact the well-established ability of mutant p53 to regulate the transcriptome of cancer cells? Second, could the drug actually change the conformation of mutant p53 such that it might regain wild-type activities? Third, does resveratrol bind to other regions in the p53 protein and thereby affect its tetramerization, activation or degradation? Finally, does resveratrol affect p53 binding to any of the myriad proteins reported to bind to p53 that could affect different cellular processes?

Resveratrol is not the only molecule that regulates p53 aggregation. Other agents have been reported to do so such as acetylcholine chloride, polyarginine and analogues [7]. As another example, a cell-penetrating peptide, ReACp53, is capable of binding to the p53 core domain and transforming aggregated mutant p53 proteins into soluble wild-type-like p53 protein [9]. Mutant p53 is stabilized and transcriptionally reactivated in the presence of ReACp53, leading to induction of cell death *in vitro* and *in vivo* and decreasing tumor size in a mouse model. Although *in vivo* studies and clinical trials have shown that resveratrol alone is more efficient as a chemopreventive than as a chemotherapeutic agent [1], when combined with established clinical protocols, resveratrol was shown to have synergistic effects in inducing tumor cell death and tumor regression [5, 10]. The ability of this drug to reduce aggregation of p53 may be relevant to its potential anti-cancer properties. Hopefully future studies will elucidate the significance of the interaction between resveratrol and p53.

## References

[1] Carter LG (2014). Endocr Relat Cancer.

[2] Ferraz da Costa DC (2018). Oncotarget.

[3] Ferraz da Costa DC (2017). Molecules.

[4] Ishimaru D (2003). Biochemistry.

[5] Kma L (2013). Asian Pac J Cancer Prev.

[6] Kraiss S (1992). Biochim Biophys Acta.

[7] Kanapathupillai M (2018). Cancers.

[8] Rangel LP (2014). Prion.

[9] Soragni A (2016). Cancer Cell.

[10] Sprouse AA (2014). Anticancer Res.

[11] Yang-Hartwich Y (2015). Oncogene.

